# Effect of continuous and intermittent electric current on lignin wastewater treatment and microbial community structure in electro-microbial system

**DOI:** 10.1038/s41598-018-34379-7

**Published:** 2019-01-28

**Authors:** Lulu Zhang, Lili Ding, Xuemeng He, Haijun Ma, Huimin Fu, Jinfeng Wang, Hongqiang Ren

**Affiliations:** State Key Laboratory of Pollution Control and Resource Reuse, School of the Environment, Nanjing University, Nanjing, 210023 Jiangsu PR China

## Abstract

In this study, complex structured soluble lignin wastewater was treated by electro-microbial system (EMS) using different direct current (DC) application modes (CR (continuous ON), IR_12h_ (12 h-ON/12 h-OFF) and IR_2h_ (2 h-ON/2 h-OFF)), and physiological characteristics and microbial communities were investigated. Results showed that CR, IR_12h_ and IR_2h_ had higher lignin removals, which were almost two times that of the control reactor (R_0_′, no current), and IR_2h_ performed best and stably. Furthermore, IR_2h_ exhibited the lowest ohmic resistance (Rs) of electrode biofilms, which could be explained by its higher abundance of electroactive bacteria. In the activated sludge of EMS, the concentration of dehydrogenase activity (DHA) and electronic transport system (ETS) in IR_2h_ were the highest (1.48 and 1.28 times of R_0_′), which contributed to its high content of adenosine triphosphate (ATP). The viability of activated sludge was not affected by different DC application modes. Phospholipid fatty acids (PLFA) analysis indicated that IR_2h_ had the maximum content of C15:1 anteiso A, C16:0 and C18:0; CR increased the content of C15:0 anteiso and decreased the content of saturated fatty acids. Genus-level results revealed that lignin-degrading bacteria, *Pseudoxanthomonas* and *Mycobacterium*, could be enriched in IR_2h_ and CR, respectively.

## Introduction

Lignin, a three-dimensional phenylpropanoid polymer^1^, is primarily responsible for the brown color in paper and pulp wastewater^2^, which is recalcitrant for most anaerobic or aerobic microorganisms during conventional biological treatment^3^. Electro-microbial system (EMS), a system coupling of electrochemical reaction and biological metabolism, can enhance the biodegradation efficiency of non-degradable materials such as phenol, *p*-fluoronitrobenzene, cefuroxime, 2,4-dichlorophenol and Alizarin yellow R by microorganisms^4–8^. However, few studies on the treatment of lignin wastewater using EMS have been reported.

Dissolved oxygen and current type (Alternating current (AC)/Direct current (DC)) play an important role in EMS when treating recalcitrant organic compounds. Among these, super high voltage AC can provide a strong electric field force and low frequency AC may generate disinfectants^9^. Hence, DC is commonly used in EMS. However, the effect of different DC application modes on the performance of EMS is not consistent. For example, when using bioelectrochemical reactors to treat phenol-containing synthetic wastewater, the phenol removal efficiency in intermittent DC mode (12 h-ON/12 h-OFF) was about 47% higher than that in the continuous one^10^. Bellagamba *et al*.^11^ found that intermittent application of electrolysis could keep the same removal rate of total petroleum hydrocarbons (TPH) as continuous mode. In contrast, it was found that the enhancement could only be realized under continuous DC mode, rather than intermittent mode (12 h-ON/12 h-OFF, 4 h-ON/4 h-OFF) when reducing nitrite^12^. Thus, DC application modes need further optimization for better performance.

It was shown that continuous and intermittent DC pattern made significant differences in bacterial counts, growth rates, microbial activity and cell membrane properties at lower current densities^13^. Intermittent current minimized the direct exposure of bacteria to the electric field, thus reducing the negative effect to microbial community^14^. Longer or more frequent time-OFF was suitable for the microorganisms to preserve their metabolic processes^15^. It is reported that applying an external electric field to cells may result in the accumulation of electrical charge at the cell membrane, which may lead to a change in voltage across the membrane^16^. As components of microbial cell membranes formed by intact and proliferating cells, PLFAs are frequently utilized to confirm the presence of active intracellular metabolism^17^. However, the above studies mostly focus on municipal wastewater and pure cultures, the physiological characteristics of microorganisms in EMS fed with recalcitrant organic wastewater need further research.

It is essential to illuminate the effect of the DC application modes on microbial structure community of activated sludge and electrode biofilms. There have been studies published relevant to the changes of microbial communities in the wastewater containing phenol, sulfate and nitrite under different DC modes. Zeyoudi *et al*.^13^ found that constantly applied DC pattern had no effect on microbial community structure, but intermittently one caused a shift in a bioreactor. Ailijiang *et al*.^10^ used bioelectrochemical reactors to treat phenol wastewater and found that genera of *Zoogloea* and *Desulfovibrio* had a significant enrichment under an intermittent DC mode. Wang *et al*.^18^ used a Microbial Electrolysis Cell (MEC) reactor to treat sulfate-rich wastewater, and found that intermittent DC field could maintain higher species diversity and *Desulfovibrio* enriched on the electrode biofilms. The above studies showed that functional bacteria could be enriched under different DC patterns. However, there have been few reports on treating lignin wastewater using EMS. The functional microbes which can degrade lignin include *Clostridium*, *Pseudomonas*, *Flavobacterium*, *Micrococcus*, *Xanthomonas*, *Mycobacterium* and *Microlunatus*, etc^19–23^. The succession of microbes mentioned in EMS above needs further study.

In this study, the effect of continuous and intermittent DC application modes on lignin wastewater treatment, electrochemical characteristics of electrode biofilms, physiological characteristics, electron transfer rate and PLFA compositions of activated sludge was studied. The differences in microbial communities of electrode biofilms and activated sludge were also investigated for a full appreciation of the effect of different DC modes on electroactive bacteria and functional microorganisms enriching.

## Results and Discussion

### Performances of Reactors

**Figure 1:**
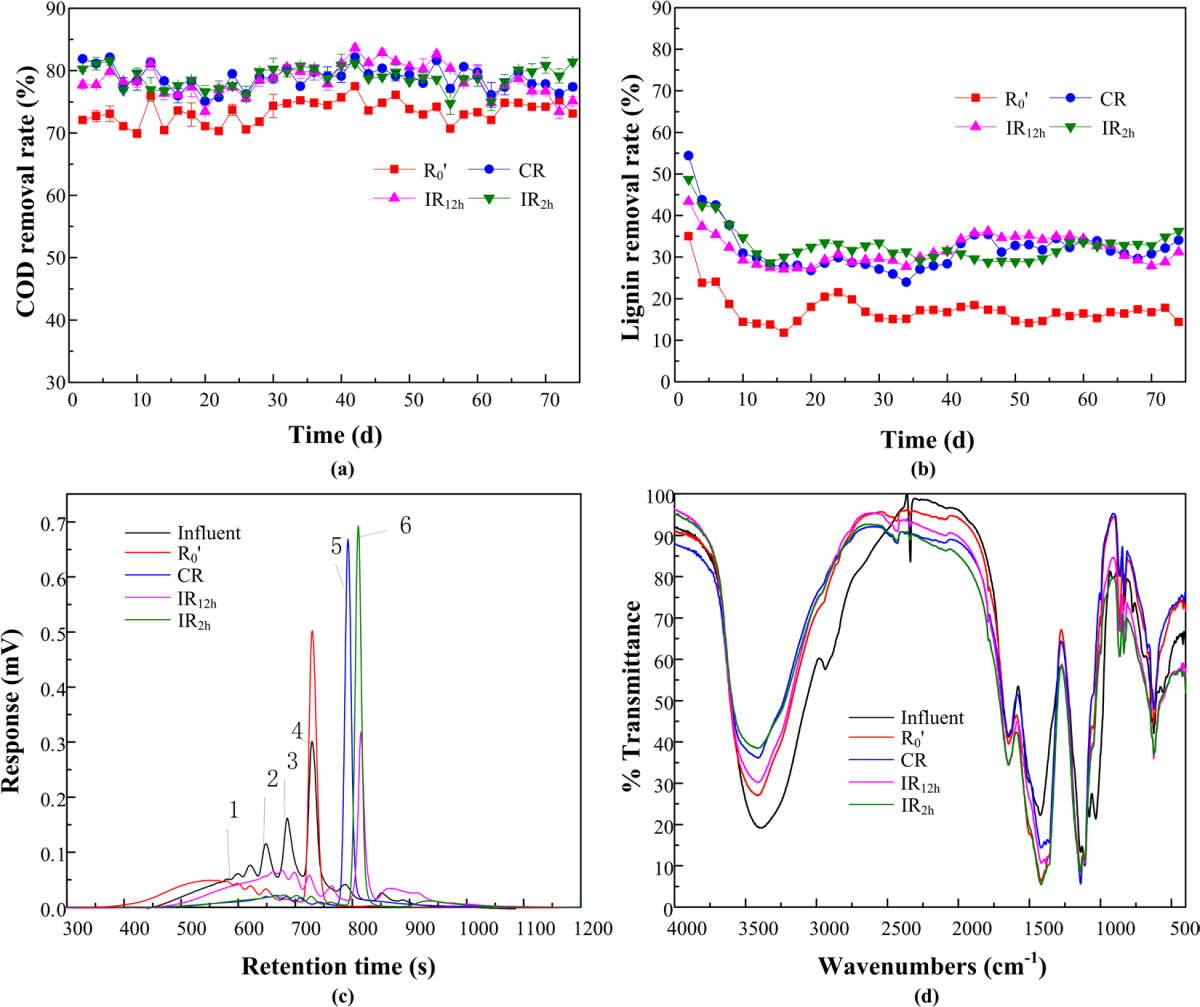
Reactor performances under different DC application modes. (**a**) COD removal rate; (**b**) Lignin removal rate; (**c**) Apparent molecular weight (AMW) distribution; (**d**) FTIR spectra.

The performances of reactors treating synthetic wastewater containing lignin were assessed (Fig. 1). During the entire operation, stable COD removal of each reactor was observed and the average COD removal rates of CR, IR_12h_ and IR_2h_ (79.04% ± 1.91%, 79.20% ± 2.58%, 79.21% ± 1.65%) were 5% higher than that of R_0_′ (74.26% ± 1.37%), indicating that different DC application modes were all able to improve COD removals under 30 mA.

The results of lignin removals were shown in Fig. 1b. The lignin concentrations in the effluents of the four reactors first decreased rapidly and then stabilized. The lignin removal rates of the four reactors dropped from 35.02%~54.42% on the 2nd day to 11.77%~30.03% on the 16th day. This means lignin was adsorbed by activated sludge in the initial period, which reached saturation on day 16, because of the excellent adsorbent properties of activated sludge and the relatively poor biodegradability of lignin^24^. Helmreich *et al*.^24^ found that the concentration of dissolved lignin in the effluent of SBR reached an approximately constant level in the rectors operated at a sludge of 20 days old and more and the observed removal is due to the adsorption by the activated sludge. It was also reported that lignin could be adsorbed on the biological flocs in the presence of activated sludge^25^. Conversely, during the same period, the removal rate of degradable substrate glucose gradually increased. Ma *et al*.^26^ reported that the SBRs fed with glucose synthetic wastewater at different DO levels improved COD removals from 75% to 90% from days 1 to 15. It can be inferred that there was a reverse removal rate of glucose and lignin in the four reactors from day 1 to day 16, which was the reason why the COD removals remained stable during the whole operation.

In the period of 17 to 75 days, the average lignin removals of IR_2h_, IR_12h_ and CR (31.75% ± 1.92%, 31.55% ± 2.82%, 30.57% ± 2.99%) were almost two times that of R_0_′ (16.34% ± 1.52%). IR_2h_ kept more stable performance, while R_0_′, CR, IR_12h_ had fluctuant lignin removals. Similarly, Ailijiang *et al*.^10^ found that a higher and more stable phenol removal efficiency could be observed under intermittent DC application mode than a continuous one with applied current of 2 mA.

The AMW distributions of influent and effluent on day 75 were shown in Fig. 1c. The MW distribution in the influent exhibited major fractions around peak 1 (1099 Da, 18.51%), peak 2 (412 Da, 12.13%), peak 3 (255 Da, 18.08%) and peak 4 (187 Da, 26.97%). After the treatment by intermittent DC patterns, high percentage of small molecular weight peak 6 (76 Da, 67.63%) was found in IR_2h_, followed by IR_12h_ (76 Da, 24.5%). In the effluent of CR, medium peak 5 (109 Da, 77.25%) was major component. In the effluent of R_0_′, the major fractions were peak 1 (1099 Da, 26.18%) and peak 4 (187 Da, 47.51%), indicating the lowest degradation efficiency.

The infrared spectra of both influent and effluent of reactors on day 75 are reported in Fig. 1d. The infrared spectra of influent showed seven peaks of 3384.6, 2938.4, 1644.8, 1423.6, 1110.3, 1032.8, 900–600 cm^−1^, corresponding to aliphatic, polysaccharide, phenyl, and aromatic structures (Table S1). This indicated that lignin structures in the influent included aromatic and phenolic structures, which were known for their absorption towards 1420, 1230 and 1130 cm^−1 ^^27^. The effluent of IR_2h_ had the weakest intensity of peak 3384.6 cm^−1^, which indicated the highest efficient degradation of phenolic structures. The significant increase in the intensity of the 1423.9 cm^−1^ of the four effluents indicated the occurrence of oxidation reactions^28^ and IR_2h_ had the strongest peak intensity. The effluents of all four reactors had a decrease in peaks at 2938.4, 1077.7 and 1032.8 cm^−1^, which could be related to the degradation of aliphatic and polysaccharides structures by the microorganisms during the treatment.

### Electrochemical characteristics

**Figure 2:**
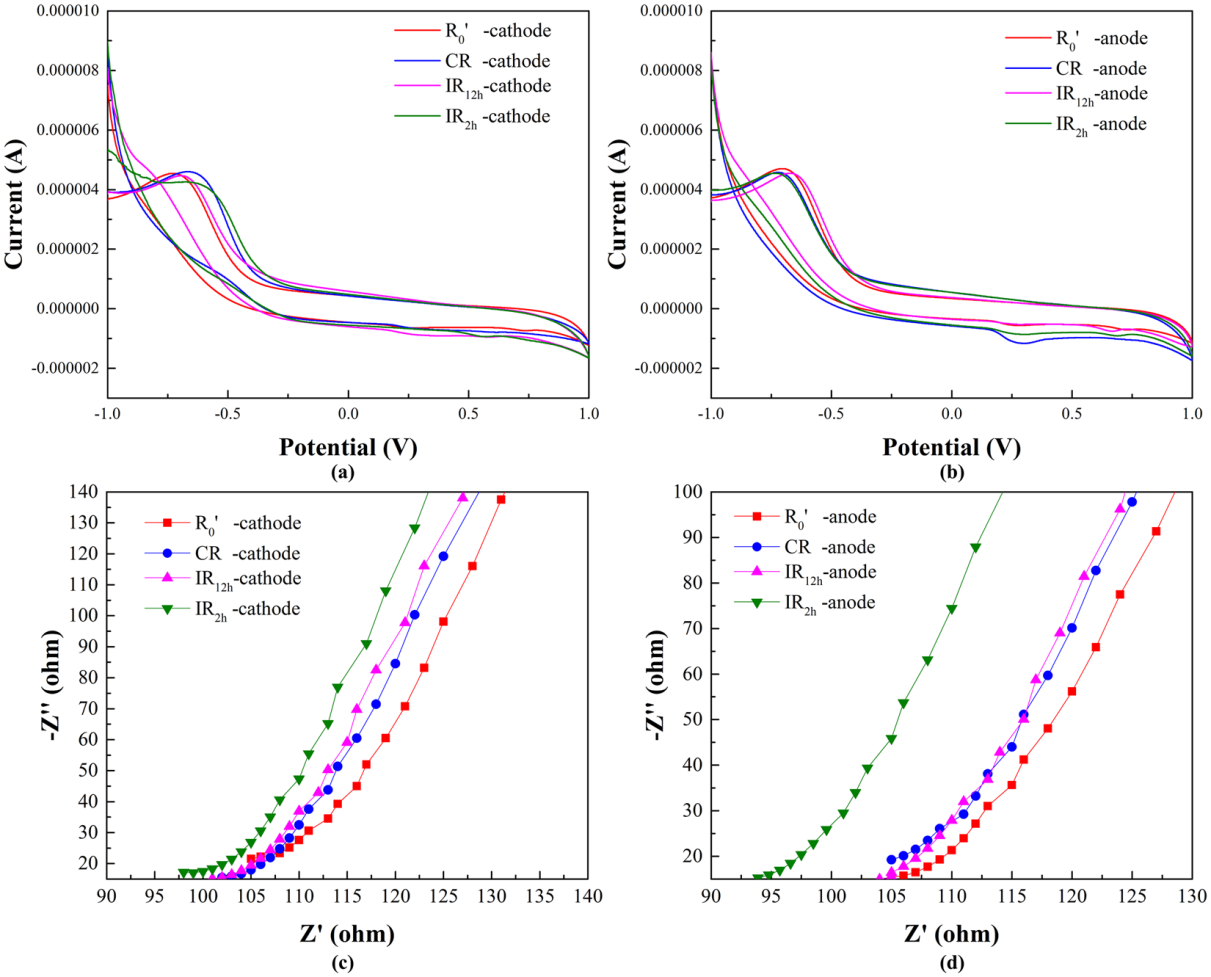
Electrochemical characterizations of the anode and cathode under different DC application modes. (**a**) Cyclic voltammograms (cathode); (**b**) Cyclic voltammograms (anode); (**c**) Electrochemical impedance spectroscopy (cathode); (**d**) Electrochemical impedance spectroscopy (anode).

The bioelectrochemical behavior of electrode biofilms on day 75 was analyzed by cyclic voltammograms (CV) shown in Fig. 2a,b. In R_0_′, no obvious irreversible oxidation peak was observed on the anodic biofilm. On the anodic biofilm of CR_,_ the position of an oxidation peak appeared at 0.3 V (vs. saturated calomel electrode (SCE)). On the anodic biofilms of IR_12h_ and IR_2h_, peaking at 0.3 V and 0.7 V, indicated the presence of electron transfer processes^29^. No significant reduction peak of the four reactors on cathodic biofilms revealed the lack of reductive compounds.

It is reported that biofilms attached to the electrode can be involved in facilitating the transfer rate of extracellular electron between electrodes and bacteria^30^. In this study, the effect of DC on the electrochemical property of electrode biofilms was investigated by electrochemical impedance spectroscopy (EIS). The results were shown in Fig. 2c,d. For both cathodic and anodic biofilms, IR_2h_ (97 Ω, 94 Ω) had the lowest charge transfer resistance. The Rs of CR (103 Ω, 105 Ω) and IR_12h_ (101 Ω, 103 Ω) were all decreased compared to R_0_′ (107 Ω, 106 Ω). It was reported that MEC reactor with intermittent DC field had a much lower charge transfer resistance than a continuous one due to the destabilization of the biofilm^18^.

### Physiological characteristics of activated sludge

#### Effect of DC modes on Viability

**Figure 3:**
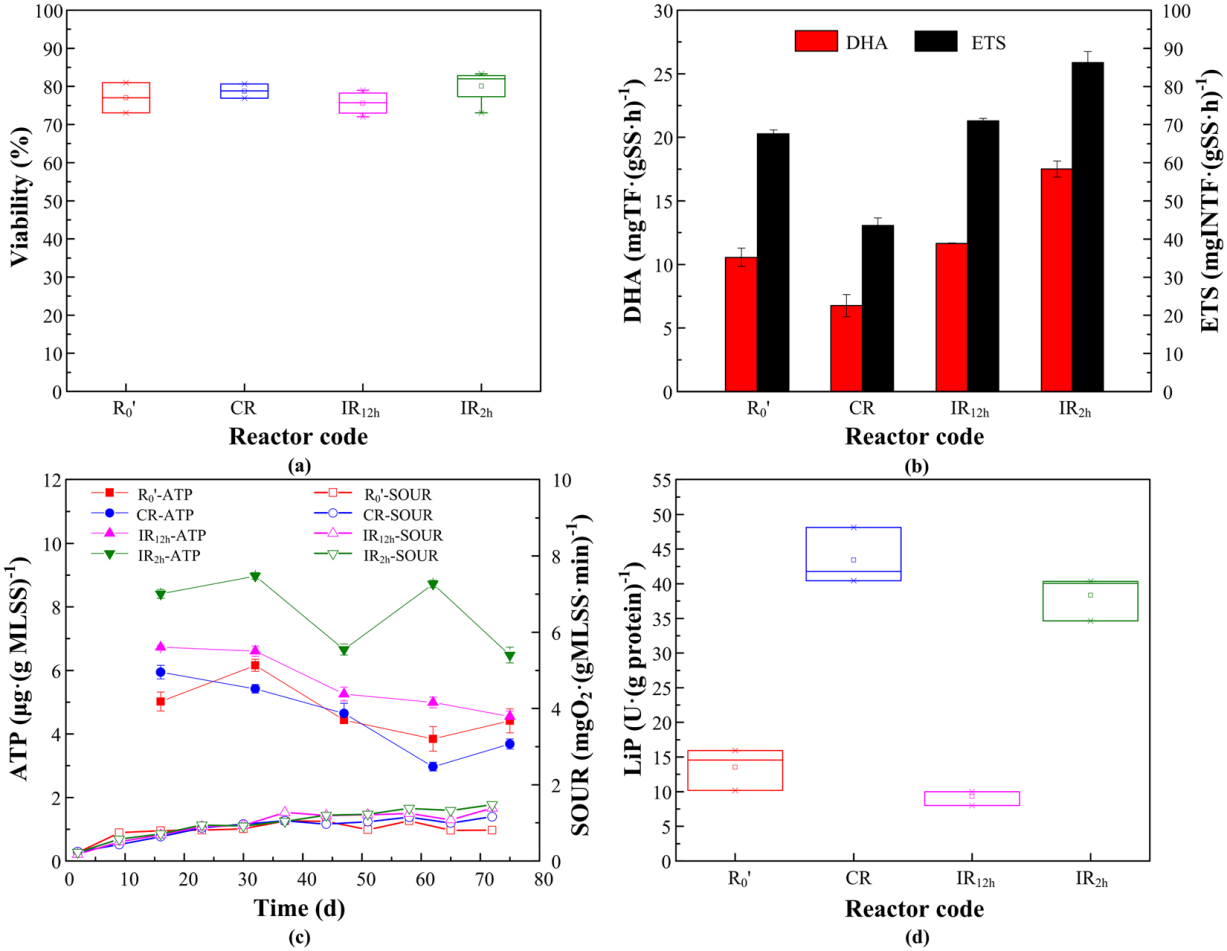
Physiological characteristics of activated sludge microorganism under different DC application modes. (**a**) Viability; (**b**) TTC-DHA and INT-ETS; (**c**) SOUR and ATP contents; (**d**) LiP activity.

LIVE/DEAD values were used to evaluate cell integrity of activated sludge after exposure to different DC application modes during the steady stage (Fig. 3a). It showed that the ratios of live bacteria of IR_2h_, CR, R_0_′ and IR_12h_ were 80.08% ± 4.09%, 78.78% ± 1.52%, 77.03% ± 3.96% and 75.62% ± 2.77%, which were almost unchanged (*p* > 0.05) under different electrical modes with 30 mA current. In the four reactors, most of the bacterial cell membranes were intact; no large amounts of bacterial apoptosis occurred in the reactor.

#### Effect of DC modes on TTC-DHA and INT-ETS

DHA catalyzed the oxidative dehydrogenation of organic compounds in biological cells and detached electrons which were eventually transferred to the final electron acceptor (O_2_, NO_3_^−^, SO_4_^2−^ etc.) through ETS; this was to convert organic pollutants into inorganic pollutants. DHA could be increased by hydrogen generated at the cathode^31^. It was evident from Fig. 3b that there was a significant correlation between TTC-DHA and INT-ETS (*p* < 0.05). Compared to the control: 10.56 ± 0.72 mgTF/(gSS·h) and 67.63 ± 0.98 mgINTF/(gSS·h), the average DHA and ETS of IR_2h_ were 17.50 ± 0.63 mgTF/(gSS·h) and 86.31 ± 2.83 mgINTF/(gSS·h), which were distinctly stimulated. IR_12h_ could maintain the bacterial activities, and the average DHA and ETS of IR_12h_ were 11.67 ± 0.03 mgTF/(gSS·h) and 71.04 ± 0.54 mgINTF/(gSS·h). While continuous DC applied mode could inhibit the DHA and ETS activities, which were 6.77 ± 0.87 mgTF/(gSS·h) and 43.60 ± 1.96 mgINTF/(gSS·h). It was reported that DC field could inactivate microorganisms in the sludge, while intermittent current was beneficial to maintain high microbial activity^15^. However, continuous application modes inhibited the DHA and ETS activity without affecting degradation performance. This may be due to electrochemical reactions, which made certain contributions to lignin degradation.

#### Effect of DC modes on ATP and SOUR

Figure 3c described the SOUR and ATP of activated sludge microorganisms in the four reactors during the steady stage. ATP concentration could be considered as an indicator of metabolic activity under DC field^32^. It is reported that ATP synthase uses the energy created by the electron transport system to convert ADP to ATP^33^. Compared to the control (4.41 ± 0.38 μg/gMLSS) at 75 days, IR_2h_ had the highest total ATP content (6.48 ± 0.25 μg/gMLSS), followed by IR_12h_ and CR (4.55 ± 0.17 and 3.68 ± 0.16 μg/gMLSS). It could be concluded that the intermittent DC application mode could not only effectively remove lignin but also maintain the metabolic activity of microorganism during the operation, and more frequent time-OFF performed better. Similar conclusion was demonstrated by Wang *et al*.^18^ that the MEC reactor using intermittent electric had the highest ATP content when degrading sulfate-rich wastewater, while continuous mode caused cell rupture and low metabolic activities.

For aerobic microorganisms, SOUR is considered to be closely related to the substrate consumption because it quantifies the oxygen needed for its oxidation^34^. SOUR is affected by the oxygen produced and consumed through the electrochemical reactions^15^. It can be seen that the three reactors under DC application modes (CR, IR_12h_, IR_2h_) had higher SOUR than R_0_′ (Fig. 3c), which were 1.43, 1.71 and 1.82 times that of R_0_′ on day 75. It was reported that when using low-degradable substrate as carbon source, short exposure at low voltages increased the oxygen consumption rate of microorganisms^34^.

#### Effect of DC modes on LiP activity

Lignin peroxidase (LiP) is one of the most common lignin-degrading enzymes. This enzyme is extracellular nonspecific and nonstereoselective, which functions together with H_2_O_2_-producing oxidases and secondary metabolites, thus, the extracellular production of H_2_O_2_ is essential^35^. In the stable stage, average LiP activities of the four reactors were shown in Fig. 3d. LiP activity of CR (42.76 ± 3.35 U/g protein) and IR_2h_ (38.34 ± 2.71 U/g protein) was sharply increased, while that of IR_12h_ (9.98 ± 1.00 U/g protein) was slightly decreased compared to the control (13.55 ± 2.39 U/g protein). Low LiP activity and the high removal rate of IR_12h_ indicated that the contributions of LiP in lignin breakdown were less under this DC applied mode.

### Effect of DC application modes on PLFAs of microbes

**Figure 4:**
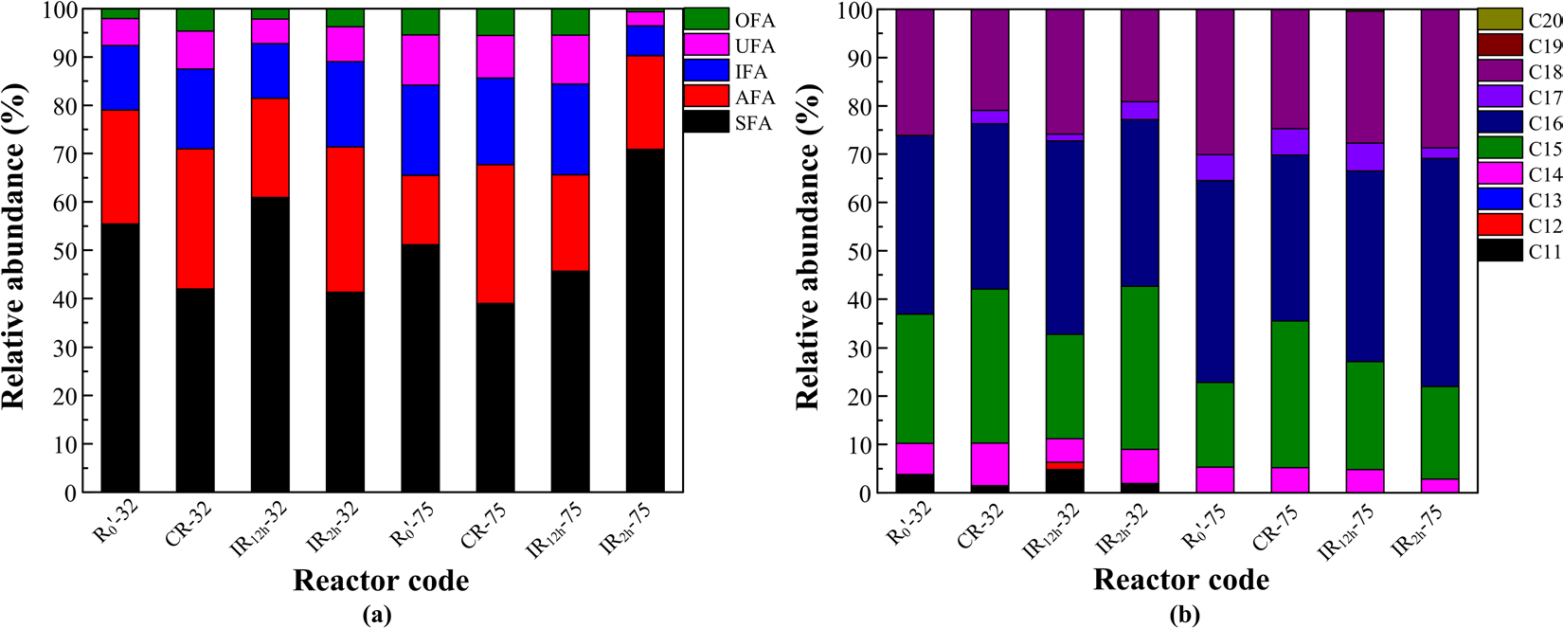
PLFA profiles of the activated sludge in four reactors on day 32 and day 75. (**a**) By structure; (**b**) By the number of carbon atoms. SFA = saturated fatty acids, AFA = anteiso-branched fatty acids, IFA = iso-branched fatty acids, UFA = unsaturated fatty acids, OFA = other fatty acids.

PLFAs, which can only be extracted from a living biomass, were used as chemotaxonomic markers and microbial stress indicators^36,37^. The components of microbial cell membranes were investigated by PLFA analysis (Fig. 4). On day 32, the total proportions of unsaturated fatty acids and breached-chain fatty acids in the four reactors were: IR_12h_ (36.9%) < R_0_′ (42.53%) < CR (53.39%) < IR_2h_ (54.98%). It was reported that branched fatty acids had the same ability as unsaturated fatty acids to disrupt the close packing of phospholipid acyl chains and lower the phase transition temperature^38^. When the four reactors ran for 75 days, the total proportions of unsaturated fatty acids and breached-chain fatty acids in the four reactors were: IR_2h_ (25.54%) < R_0_′ (33.04%) < IR_12h_ (38.75%) < CR (46.67%). It can be seen that IR_2h_ decreased the total proportions of unsaturated fatty acid and branched fatty acid, and increased the proportion of saturated fatty acid. It is reported that cells produce more saturated fatty acids when adapting to an environment that requires more rigidity in membrane lipids. When more fluidity is needed, more unsaturated or branched-chain fatty acids are produced^39^.

On day 32 and day 75, the major branched fatty acids (abundance > 1%) of the four reactors were C15:0 anteiso, C14:0 iso, C15:0 iso, C16:0 iso and C17:0 anteiso (Table S2). Notably, it can be seen that IR_2h_ had the highest content of C16:0 on day 75, which is reported to have a positive correlation between biomass^40^. In addition, the proportion of C15:0 anteiso increased significantly in CR (23.58%) in comparison to the other reactors (11.36%, 15.8%, 15.42%) on day 75. It was found that C15:0 anteiso had the lowest temperature of phase transition (23 °C)^26,41^ to maintain better cell membrane fluidity for microbes^26^. These results showed that with DC application modes of continuous ON, microbes could increase cell membranes fluidity to achieve optimal growth by increasing the content of C15:0 anteiso.

### Effect of DC application modes on microbial communities

#### Activated sludge

The microbial community structure under different DC application modes on day 32 and day 75 were analyzed (Fig. S1a). It can be seen that the microbial community at phylum level in R_0_′ dominantly consisted of Proteobacteria (23.78%), Actinobacteria (33.06%), Bacteroidetes (11.96%) and TM7 (9.05%) on day 32. It is reported that there are many lignin-degrading aerobic bacteria mainly in Actinobacteria, α-Proteobacteria and γ-Proteobacteria^42^. The relative abundance of Proteobacteria in CR, IR_12h_ and IR_2h_ (25.71–27.97%) was slightly higher than that in R_0_′ (23.78%), especially intermittent modes. When the reactors ran for 75 days, the microbial community had changed greatly (Fig. S1a). The relative abundance of Proteobacteria decreased and the relative abundance of Actinobacteria increased significantly in the four reactors. Actinobacteria had the ability to degrade solubilize lignin and some cellulose, and were important agents of lignocellulose degradation^43^. The relative abundance of TM7 in CR, IR_12h_ and IR_2h_ (4.14%, 2.62%, 10.07%) were all lower than R_0_′ (21.02%), suggesting that the electricity environment was not suitable for the growth of TM7. A similar phenomenon was found by Yu *et al*.^44^. The relative abundance of Bacteroidetes decreased, and there was little difference between the four reactors (3.14%, 3.94%, 5.85%, 4.68%), indicating that Bacteroidetes was not affected by the DC field. It was reported that Bacteroidetes exerted an enormous function on the use of protein and chitin, and degraded some high molecular weight of the dissolved organic matter (DOM) proficiently^26^.

**Figure 5:**
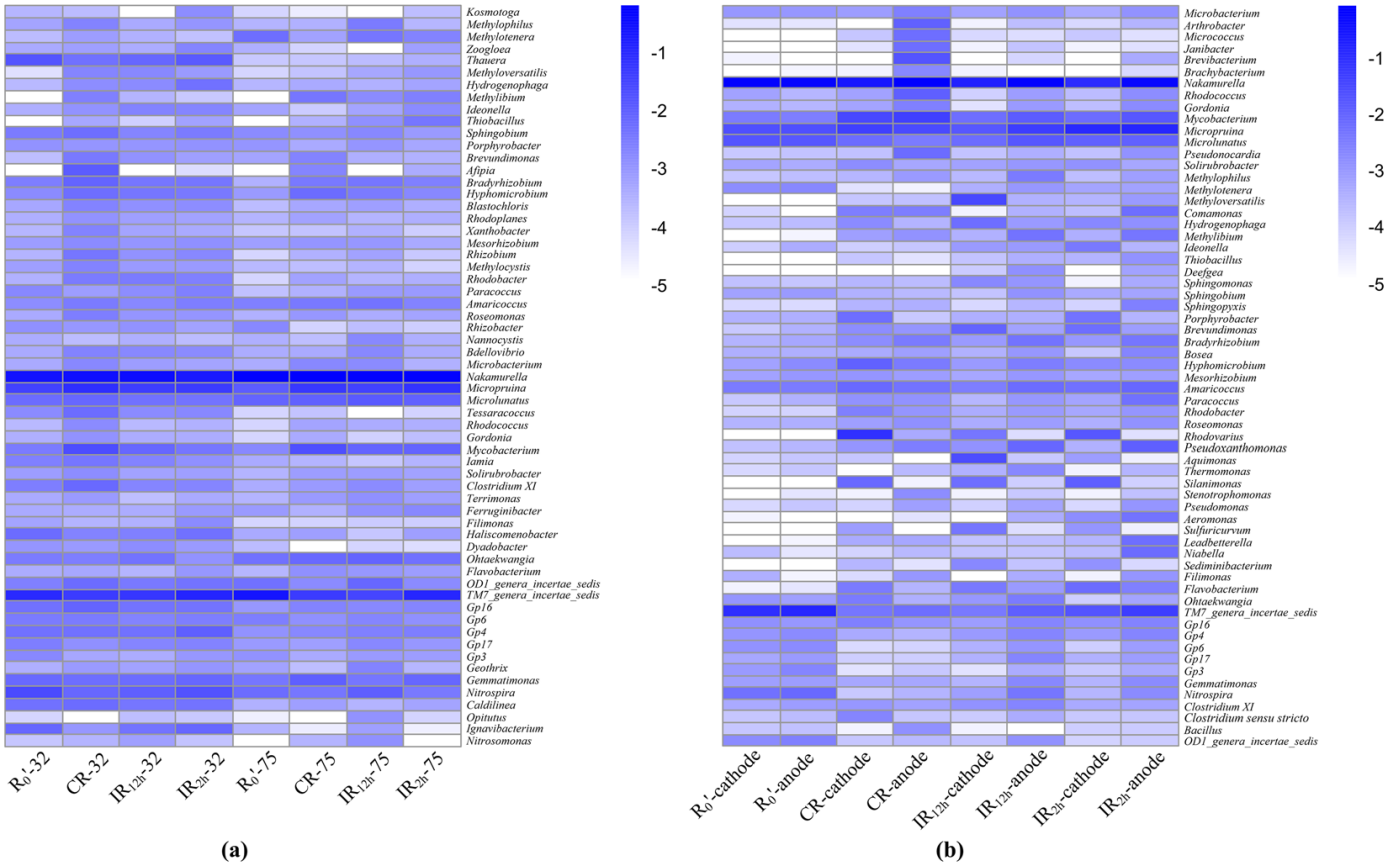
Heat map of genera (abundance > 0.20%) of microorganisms in SBRs under different DC application modes. (**a**) Activated sludge; (**b**) Electrode biofilms.

Figure 5a is the heat map of microbial community at the genus level (>0.2%). The relative abundance at the genus level changed greatly in the four reactors from the 32nd day to 75th day. Figure 5a showed that *Nakamurella*, *TM7_genera_incertae_sedis* and *Micropruina* were consistently abundant in the sludge of four reactors on the 32nd day. On day 75, the microbial communities had a great change and the dominant genus in R_0_′, CR, IR_12h_ and IR_2h_ was *Nakamurella*, which had a significant increase. In CR, IR_12h_ and IR_2h_ (4.76%, 3.05%, 6.05%), the relative abundance of *Micropruina* were all higher than that in R_0_′ (1.00%). It has been reported that *Nakamurella* and *Micropruina* were able to accumulate energy-storage chemicals in response to harsh conditions^45^. In CR, IR_12h_ and IR_2h_, the content of *TM7_genera_incertae_sedis* all had a slight decrease (4.14%, 2.62%, 10.07%) but it increased distinctly in R_0_′ (21.02%) on the 75th day, which has been reported commonly present in activated sludge^46^. Compared to R_0_′, lignin-degrading bacteria, *Mycobacterium* and *Microlunatus*^23^, were enriched in sludge under continuous and intermittent modes, respectively.

#### Electrode biofilms

Microbial communities on electrode biofilms under different DC application modes were analyzed. Figure S1b shows the relative abundance at the phylum level. The microbial community structure of electrode biofilms of R_0_′ can be seen as a parallel control group without the DC field. Actinobacteria were the predominant phylum with relative abundance ranging from 69.43% to 76.16%. TM7, Proteobacteria and Bacteroidetes were the subdominant groups, comprising 9.44% to 12.23%, 7.63% to 10.51%, and 1.06% to 1.26% of the detections, respectively. In cathodic biofilm, the abundance of Proteobacteria in CR, IR_12h_ and IR_2h_ (57.19–73.32%) increased obviously compared to R_0_′ (7.63–10.51%). Many studies have demonstrated that electrogenic bacteria were widely distributed in Proteobacteria^44,47^. The abundance of Actinobacteria and TM7 in CR, IR_12h_ and IR_2h_ (22.58–36.73%, 0.37–1.65%) decreased obviously compared to R_0_′ (69.43–76.16%, 9.44–12.23%), respectively. In anodic biofilm, the abundance of Proteobacteria of IR_2h_ (16.63%) was the highest, which could transfer electron between bacteria and electrode^48^. The abundance of Actinobacteria in CR (89.22%) increased obviously compared to R_0_′ (69.43–76.16%), and a similar phenomenon was found by Wang *et al*.^30^ that the phylum Actinobacteria was enriched in the electrode biofilms of MEC reactor under continuous mode. In both cathodic and anodic biofilms, the abundance of Bacteroidetes in CR, IR_12h_ and IR_2h_ (0.65–3.27%) was almost unchanged in comparison with R_0_′ (1.06–1.26%), which was similar to the phenomenon in activated sludge. These results showed that electrode biofilms exposed to different DC application modes differed in abundance of microbial community at the phylum level.

Figure 5b is the heat map of microbial community including 63 most abundant genera at the genus level. Four genera were abundant (>1%) in electrodes of R_0_′, including *Nakamurella* (54.84–60.79%), *TM7_genera_incertae_sedis* (9.44–12.23%), *Micropruina* (2.08–2.17%) and *Microlunatus* (1.44–1.66%). The relative abundance of *Pseudoxanthomonas* in anodic biofilms of IR_2h_ (1.08%) and IR_12h_ (0.78%) was obviously higher than that in other samples (0.01%-0.27%); this was considered as a lignin-degrading bacteria and was commonly present in lignocellulose degradation^49^. The relative abundance of *Micropruina* of IR_2h_ (11.39–12.95%) was obviously higher than that in other samples (2.08–2.17%, 2.69–4.02%, 1.47–4.57%, respectively), which could tolerate some toxicity^50,51^. It was reported that *Micropruina* was positively correlated with effluent qualities and microbial activity^52^. However, the relative abundance of *Nakamurella* in the cathodic biofilms of CR, IR_12h_ and IR_2h_ (9.92–25.27%) was lower than that in R_0_′ (54.84–60.79%). The relative abundance of *TM7_genera_incertae_sedis* was obviously lower in biofilms of CR, IR_12h_ and IR_2h_ (0.37–4.80%) than that in R_0_′ (9.44–12.23%). While the role of *TM7_genera_incertae_sedis* might play in the aerobic wastewater treatment was unclear^46^. Moreover, the highest relative abundance of *Aeromonas* detected in anodic biofilm of IR_2h_ (0.43%), which was reported to be an electroactive microorganism^53^, may result in the lowest ohmic resistance (Rs) of electrode biofilm.

### RDA analysis of microbial community with effluent qualities, physiological characteristics and PLFA composition

**Figure 6:**
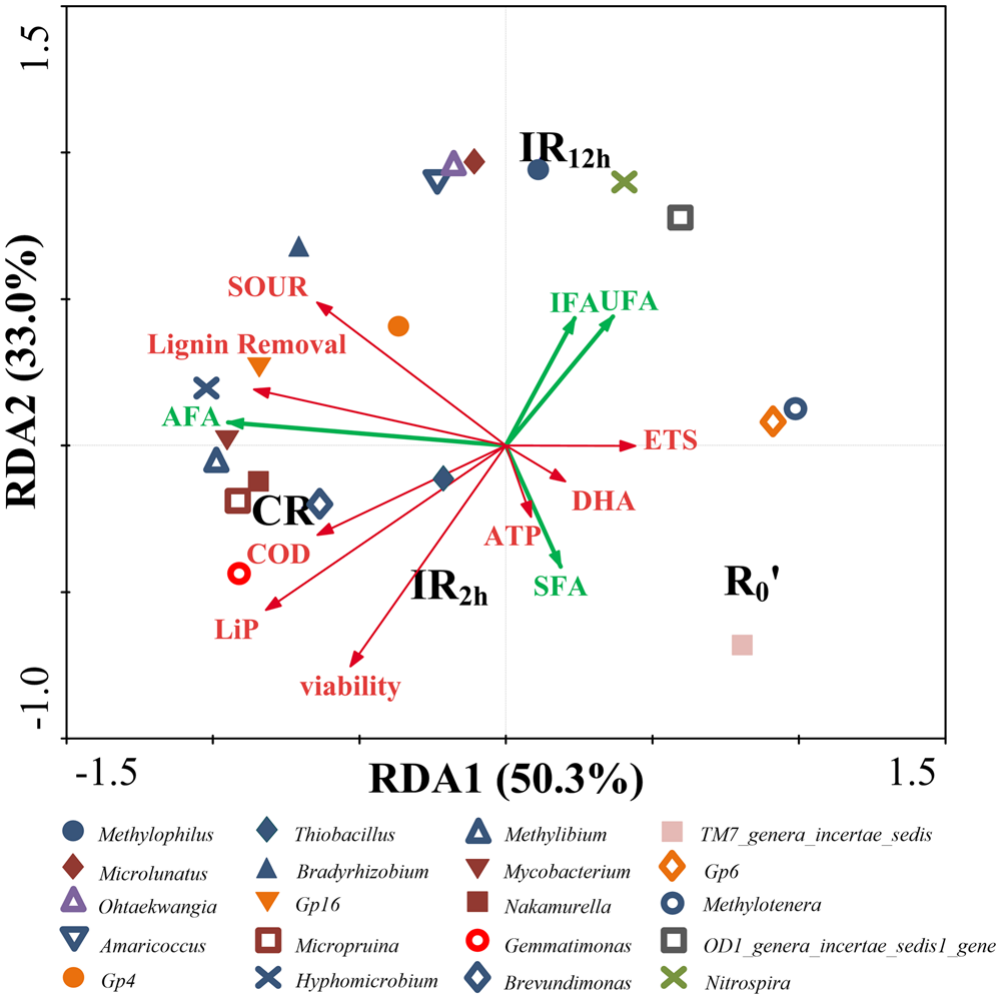
RDA analysis of microbial community in relation to phospholipid, effluent quality and physiological characteristics of activated sludge microorganism.

RDA Analysis was shown in Fig. 6, with RDA1 explained 50.3%, and RDA2 explained 33.0% of the total variance, respectively. Effluent qualities and physiological characteristics were positively correlated with *Methylibium*, *Thiobacillus*, *Bradyrhizobium*, *Hyphomicrobium*, *Nakamurella*, *Micropruina*, *Microlunatus*, *Mycobacterium*, *Gp16*, *Gp4* and *Gemmatimonas*. Bacteria mentioned above belong to Proteobacteria, Actinobacteria, Acidobacteria and Gemmatimonadetes and the abundances of Proteobacteria and Actinobacteria were relatively high. According to Pearson correlation analysis, it was found that *Micropruina* showed high positive correlation with COD removals (*p* < 0.05) and *Gemmatimonas* showed high positive correlation with LiP (*p* < 0.05). However, there was no microbial population significantly correlated to saturated fatty acids (SFA), unsaturated fatty acids (UFA) and iso-fatty acids (IFA) (*p *> 0.05). *Hyphomicrobium* and *Mycobacterium* were positively correlated with anteiso-fatty acids (AFA) (*p* < 0.05). Furthermore, IR_2h_ had positive correlation with viability, and negative correlation with IFA and UFA, which was significantly distinct with IR_12h_. This means current modes lead to the difference of physiological characteristics and microbial structure. CR was negatively correlated with ETS and DHA, which means continuous DC mode might deter electron transfer. The above results revealed that different DC application modes promoted the physiological characteristics of activated sludge, the changes of PLFA compositions and the shifts of microbial community, which influenced the effluent quality.

## Conclusion

This study demonstrated that both intermittent and continuous DC application modes could obviously increase lignin removal efficiency and intermittent application modes were prior to the continuous one, especially 2 h-ON/2 h-OFF mode. Smallest molecules in effluent, best sludge activity and fastest extracellular electron transfer rate could be achieved under 2 h-ON/2 h-OFF mode. Furthermore, IR_2h_ exhibited the lowest ohmic resistance (Rs) of electrode biofilms (97 Ω, 94 Ω) due to its highest abundance of electroactive bacteria. Microbial community analysis revealed that functional microbes for lignin degrading were enriched under different DC application modes.

These conclusions reveal that low intermittent electrical current coupling to activated sludge process promoted the treatment of recalcitrant wastewater with higher electron transfer rate and lower resistance and energy consumption. EMS provides an attractive alternative to industries for wastewater treatment, such as paper and pulp wastewater, phenol-containing wastewater and dye-containing wastewater. Moreover, in addition to wastewater treatment, EMS has a potential utilization in other fields, such as metal recovery and functional material synthesis based on microbial-electrochemical metabolism^54^. In the future, more efforts will be needed to make on high-efficiency anti-corrosion electrode materials and the electron transfer mechanisms of intermittent DC applied mode, especially microbial nanowires and electron transfer mediators in the biofilm of electrodes.

## Materials and Methods

### Experimental start-up

Four parallel SBRs (R_0_′, CR, IR_12h,_ IR_2h_) were established in the laboratory scale; the total and effective volume was 2.5 and 2 L, respectively. Each reactor was installed with a pair of graphite electrodes which had an effective area of 60 cm^2^ (20 cm × 5 cm × 6 mm) and were inserted with a distance of 7 cm between the electrodes. The electrodes in CR (continuous ON), IR_12h_ (12h-ON/12 h-OFF), IR_2h_ (2 h-ON/2 h-OFF) were supplied with a regulated DC power source (PS-305DF, China) with a constant current of 30 mA, and R_0_′ was a control with no electric field applied.

The reactors were inoculated with seed sludge collected from Dachang Sewage Plant in Nanjing, China. The MLSS concentrations in the four reactors were about 3000 mg/L. One liter of synthetic wastewater contained 300 mg of glucose, 200 mg of sodium lignosulfonate, 117.2 mg of NH_4_Cl, 26.9 mg of KH_2_PO_4_, yielding an influent chemical oxygen demand (COD) of 530 ± 10 mg/L. The synthetic wastewater also contained: MgSO_4_·7H_2_O 0.055 g·L^−1^, CaCl_2_·2H_2_O 0.025 g·L^−1^, FeCl_3_·6H_2_O 1.5 g·L^−1^, KI 0.18 g·L^−1^, ZnSO_4_·7H_2_O 0.12 g·L^−1^, H_3_BO_3_ 0.15 g·L^−1^, MnCl_2_·4H_2_O 0.12 g·L^−1^, CoCl_2_·6H_2_O 0.15 g·L^−1^, CuSO_4_·5H_2_O 0.03 g·L^−1^, Na_2_MoO_4_·2H_2_O 0.06 g·L^−1^, and EDTA-4Na 10 g·L^−1^ as trace nutrients. The reactors were operated following a 24 h cycle. The time of feeding, aeration, settling and withdrawing was 30, 1320, 60, and 30 minutes, respectively. The reactors were operated at room temperature (25 ± 2 °C) and the initial pH was adjusted to 7.6 ± 0.1 (FE20, METTLER TOLEDO Inc., USA).

### Electrochemical characterizations

To evaluate the performance of oxygen reduction reaction of electrodes, the cyclic voltammograms (CV) were systematically performed by a potentiostat (CHI660E, Chenhua Instruments Co. Ltd., Shanghai, China) at a scan rate of 20 mV/s between −1.0 V and 1.0 V in the 10 mM phosphoric acid buffer solution (pH = 7.4). The electrochemical system was a three-electrode system. Hg/HgCl_2_ (saturated KCl) was used as a reference electrode and a platinum sheet (1 cm^2^) was used as a counter electrode. The working electrodes were the electrodes of the reactors. The voltages in the text were measured with Hg/HgCl_2_ as a reference electrode. Electrochemical impedance spectroscopy (EIS) was measured over the frequency range of 0.01 Hz-100 kHz and the amplitude was 10 mV. Tests of electrodes were kept under the same conditions^55^.

### Physiological characteristics

Lignin peroxidase (LiP) activity in activated sludge was determined according to Rajwar *et al*.^56^. The viability and changes of bacterial cellular permeability in activated sludge were analyzed by fluorescent staining using a LIVE/DEAD BacLight Bacterial Viability Kit (Invitrogen Molecular Probes, USA)^9^. The method of determining ATP content was described by Ailijiang *et al*.^10^. Specific oxygen uptake rate (SOUR) was measured by the method according to Hao *et al*.^57^. The effects of DC application modes on the DHA and ETS activity of sludge microorganisms were determined by the method according to Feng *et al*.^58^.

### PLFA analysis

The method of extract PLFA was described by Niu *et al*.^59^. After a series of pretreatments, such as extraction, separation, saponification, methylation, extraction and lavation, the extracted PLFA was determined using Agilent 7890 GC^26^.

### Microbial analysis

Sixteen samples were collected during the experiment. DNA of these samples was extracted using a FastDNA Spin Kit for Soil (MP Biomedicals, Santa Ana, CA). The V1V2 hypervariable region of the 16 S rRNA gene was amplified using twenty-four different pairs of bacterial primers with different 8-base barcodes and a Guanine: 8 F (5′-AGAGTTTGATYMTGGCTCAG-3′) and 338 R (5′-TGCTGCCTCCCGTAGGAGT-3′), which were linked to the 5′ end of each primer^50^. The acquired data was processed according to the method described by Ma *et al*.^26^

### Analytical methods

COD was measured according to standard methods^60^. For determining sodium lignosulfonate in wastewater, influent and effluent samples were collected from each reactor and filtered through a 0.45 μm pore-size filter (Xinya, Shanghai, China). Contents of sodium lignosulfonate were measured at 276 nm by UV spectrophotometer (UV-2450)^61^. Infrared spectrum was determined using Fourier infrared spectrometer. The apparent molecular weight (AMW) distribution of flax wastewater was determined using the method established by Fan *et al*.^62^.

### Data analysis

The results were expressed as means ± standard deviation (SD). RDA based on the community composition, PLFA compositions, physiological characteristics and effluent quality were performed using CANOCO 4.5 software. One-way analysis of variance (AVONA) was performed with SPSS statistics 22.0.0 to evaluate whether different species had significant differences for various environmental factors. A *p* value of <0.05 was accepted as indicating significance.

## Electronic supplementary material

Supplementary Information for: Effect of continuous and intermittent electric current on lignin wastewater treatment and microbial community structure in electro-microbial system
