# Supplementary material for: Effect of continuous and intermittent electric current on lignin wastewater treatment and microbial community structure in electro-microbial system
Source: Sci Rep. 2019 Jan 28;9:805. doi: 10.1038/s41598-018-34379-7 (PMC6349836; doi:10.1038/s41598-018-34379-7)
Supplement: Supplementary file 1 — Supplementary Information for: Effect of continuous and intermittent electric current on lignin wastewater treatment and microbial community structure in electro-microbial system [file 41598_2018_34379_MOESM1_ESM.docx]

**Supplementary Information for:**

**Effect of continuous and intermittent electric current on lignin wastewater treatment and microbial community structure in electro-microbial system**

Lulu Zhang∇, Lili Ding∇, Xuemeng He, Haijun Ma, Huimin Fu, Jinfeng Wang, Hongqiang Ren*

**State Key Laboratory of Pollution Control and Resource Reuse, School of the Environment, Nanjing University, Nanjing 210023, Jiangsu, PR China**

***Corresponding authors: Hongqiang Ren, Tel: +86 25 89680512, Fax: +86 25 89680569, E-mail address:** [**hqren@nju.edu.cn**](mailto:hqren@nju.edu.cn)

∇Lulu Zhang and Lili Ding contributed equally to all aspects of conceptualizing planning, sample and data collection, data analysis and preparation of the manuscript.

This document consists of 5 pages, 2 tables, 1 figure and reference.

**Table S1:** IR absorbance bands of influent and effluents.

**Table S2:** The whole PLFA profiles of activated sludge under different DC application modes (abundance > 1%).

**Figure S1:** Abundances of different phylum (abundance>0.10%) of microorganisms in SBRs under different DC application modes. (a) Activated sludge; (b) Electrode biofilms.

# Table S1: IR absorbance bands of influent and effluents^1^

| Wavenumber (cm^-1^) | Correspondence |
| --- | --- |
| 3550–3300 | Vibration of elongation (n) H–OH (phenols, alcohols, and carboxylic groups), N–H (amides and amine) |
| 2920 | Vibration of elongation C–H of aliphatic structure (fatty acids, waxes, and various aliphatic components) |
| 1600-1620 | Vibration of elongation C=O of amides I  Vibration of elongation C=O ketones, acids, and/or quinoids  Vibration of elongation C=C aromatic |
| 1460 | Vibration of elongation C–H of aliphatic structure  Vibration of elongation C=C aromatic |
| 1120 and 1030–1050 | Vibration of elongation C–C aliphatic  Vibration of elongation C–O polysaccharides, C–O of ether on aromatic cycle |
| 600–900 | Aromatic groups |

# Table S2: The whole PLFA profiles of activated sludge under different DC application modes (abundance > 1%)

| Peak Name | R_0_'-32 | CR-32 | IR_12h_-32 | IR_2h_-32 | R_0_'-75 | CR-75 | IR_12h_-75 | IR_2h_-75 |
| --- | --- | --- | --- | --- | --- | --- | --- | --- |
| C11:00 | 3.81 | 1.48 | 4.79 | 1.94 | ND | ND | ND | ND |
| C12:00 | ND | ND | 1.53 | ND | ND | ND | ND | ND |
| C14:0 iso | 4.38 | 4.8 | 2.99 | 4.92 | 2.88 | 3.33 | 2.88 | 1.49 |
| C14:00 | 2.04 | 4.02 | 1.89 | 2.14 | 2.43 | 1.9 | 1.92 | 1.31 |
| C15:1 anteiso A | 2.43 | 3.53 | 1.89 | 4.05 | 1.49 | 2.42 | 2.21 | 2.66 |
| C15:0 iso | 1.99 | 2.51 | 2.48 | 3.52 | 3.36 | 3.03 | 3.02 | 0.6 |
| C15:0 anteiso | 21.12 | 24.07 | 17.24 | 24.55 | 11.36 | 23.58 | 15.8 | 15.42 |
| C15:00 | 1.1 | 1.69 | ND | 1.5 | 1.33 | 1.19 | 1.19 | 0.52 |
| C16:0 iso | 6.99 | 9.16 | 5.84 | 8.38 | 10.61 | 10.48 | 10.83 | 4.05 |
| C16:1 cis 9 | ND | 2.06 | 1 | 2.84 | 1.56 | 1.36 | 1.81 | ND |
| C16:00 | 30 | 22.99 | 33.1 | 22.55 | 28.21 | 22.49 | 25.29 | 43.09 |
| C16:0 iso 2OH | ND | ND | ND | ND | 0.86 | ND | 1.01 | ND |
| C17:0 iso | ND | ND | ND | 0.84 | 0.98 | 1.05 | 0.88 | ND |
| C17:0 anteiso | ND | 1.42 | 1.4 | 1.51 | 1.5 | 2.3 | 1.97 | 1.32 |
| C17:1 cis 9 | ND | ND | ND | ND | 1.18 | 0.64 | 1.06 | ND |
| C17:00 | ND | 1.36 | ND | 1.36 | 1.21 | 1.49 | 1.26 | 0.86 |
| C18:1 cis 9 | 5.62 | 5.84 | 4.06 | 3.6 | 7.2 | 6.81 | 6.86 | 2.93 |
| C18:00 | 18.49 | 10.45 | 19.62 | 11.78 | 17.94 | 11.88 | 15.55 | 25.1 |
| C18:0 anteiso | ND | ND | ND | ND | ND | ND | ND | ND |
| C18:0 10methy, TBSA | ND | ND | ND | ND | 2.12 | 2.24 | 2.18 | ND |
| C18:1 trans 9/t6/c11 | 2.02 | 4.64 | 2.16 | 3.73 | 2.84 | 3.33 | 2.71 | 0.65 |

^*^The values were the percentage of the peak area of each analyte to the total area of each sample. ND represents “none detected”.

**
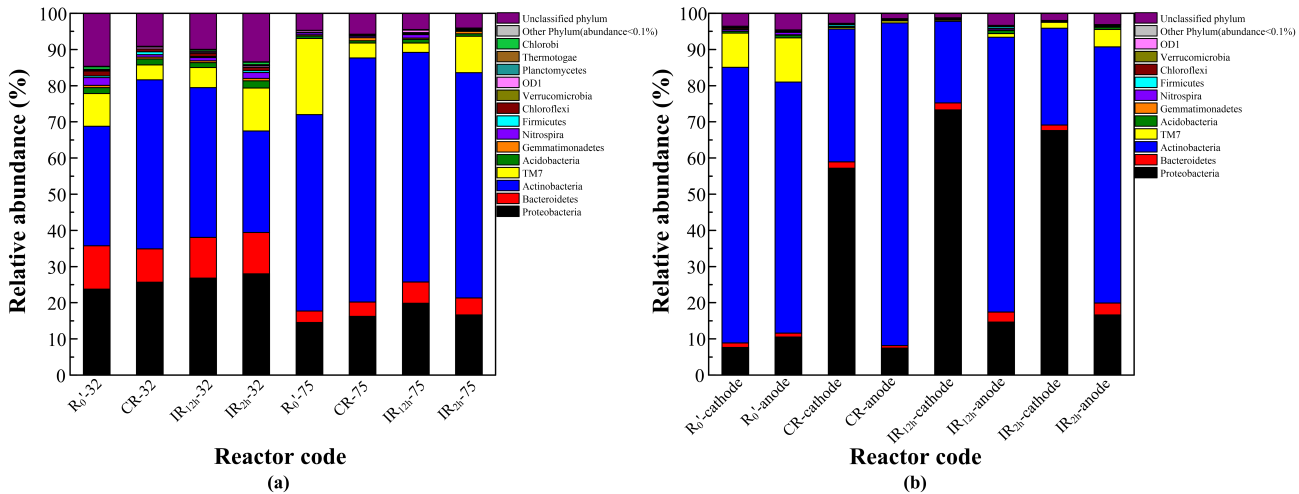
**

# Figure S1: Abundances of different phylum (abundance>0.10%) of microorganisms in SBRs under different DC application modes. (a) Activated sludge; (b) Electrode biofilms.

**References:**

1 El Ouaqoudi, F. Z. *et al.* Study of humic acids during composting of ligno-cellulose waste by infra-red spectroscopic and thermogravimetric/thermal differential analysis. *Compost Sci. Util.* **22**, 188-198 (2014).
